# Analysis of heterogeneity of the different health technology assessment reports produced on the transcatheter aortic valve implantation in patients with severe aortic valve stenosis at low surgical risk

**DOI:** 10.3389/fcvm.2023.1204520

**Published:** 2023-08-10

**Authors:** Filippo Rumi, Agostino Fortunato, Debora Antonini, Ludovica Siviero, Americo Cicchetti

**Affiliations:** Alta Scuola di Economia e Management dei Sistemi Sanitari (ALTEMS), Università Cattolica del Sacro Cuore, Rome, Italy

**Keywords:** heterogeneity of HTA, health technology assessment, TAVI, HTA methods, severe aortic valve stenosis

## Abstract

**Background:**

Symptomatic severe aortic stenosis is a congenital or acquired aortic valve disease that occurs when the aortic valve of the heart narrows. It represents the most common valvular disease in adults and generally has a degenerative nature. Transcatheter aortic valve implantation (TAVI), due to its non-invasive approach, has become the standard treatment in patients who are ineligible to surgery or at high surgical risk, and it is also increasingly being performed in patients at intermediate to low surgical risk. The aim is to analyze the heterogeneity and explore the limitations of current health technology assessments (HTAs) on TAVI.

**Methods:**

For the purpose of this analysis, a review of the literature based on manual research was performed. A population, intervention, comparators, and outcome (PICO) model was used to gather the HTA reports assessing TAVI in the treatment of patients affected by symptomatic severe aortic valve stenosis at low surgical risk. Furthermore, a manual search has been developed to also include assessments from the Haute Autorité de Santé.

**Results:**

At the end of the investigation, a certain degree of heterogeneity in the evidence factored and in the recommendations on the technology has emerged. Relative to the clinical domains, the main drivers for the disparity are found in the type of evidence considered and in the use or not of the grading of recommendations, assessment, development, and evaluation (GRADE) methodology to evaluate the quality of the clinical evidence included. Another element concerns the chosen device generation assessed within the evaluation. In order to perform the economic evaluation, a cost-utility analysis and a budget impact model were developed. Despite some elements of heterogeneity, the economic assessments demonstrate a favorable or dominant cost-effectiveness profile for TAVI compared with surgical aortic valve replacement (SAVR).

**Conclusion:**

Despite the presence of heterogeneity elements both in clinical and economic domains, HTA agencies reached the same recommendations on the use of TAVI. It emerged the need for a centralized vision on the “strong” domains, which means giving up freedom to local bodies to adapt to their context on the “soft” ones. This approach could have the potential to strengthen the role of HTA in Europe by ensuring faster decision-making and equity of access to health innovations and reduce the heterogeneity in the assessment methods.

## 1. Background

Transcatheter aortic valve implantation (TAVI) has become the standard treatment in patients who are ineligible to surgery or at high surgical risk, and it is also increasingly being performed in patients at intermediate to low surgical risk. In the ESC/EACTS 2021 guidelines recently published, TAVI is considered the standard of care even for patients with 75 years of age where the transfemoral approach is feasible (Class recommendation 1A) (1). This underlines a certain tendency to go beyond the surgical risk criteria, assuming that future evaluations will take all patients into account by considering other criteria such as age and feasibility of transfemoral access approach. Currently, the literature on TAVI is extensive and well-established, particularly in terms of its efficacy, safety, and economic outcomes in the elderly population over the mid- to long-term. As a result, TAVI has become a widely utilized technology and a standard of care in clinical practice for patients with symptomatic severe aortic stenosis (sSAS). The sSAS is the most prevalent native heart valve disease among adults in Europe, with its occurrence rate strongly associated with population aging. It is expected to pose an increasingly significant public health challenge in the future, given its degenerative nature resulting from age-dependent calcium accumulation in the aortic valve (2, 3). TAVI, as compared with conventional surgical aortic valve replacement (SAVR), is a less invasive procedure for patients with severe aortic valve stenosis, regardless of surgical risk. TAVI is achieved through transcatheter access to the aortic valve, with options such as transfemoral (TF), transapical (TA), or transaxillary/subclavian routes. Among these, TF access is the most common and least invasive route in clinical practice. TAVI devices were initially CE marked in 2007 for patients deemed inoperable or at high risk for surgical complications or death due to symptomatic severe aortic stenosis. In 2019, its use was expanded to include low-risk patients. A recent overview by Hawlik et al. (4) revealed that 22 health technology assessment (HTA) reports were conducted in Europe to evaluate TAVI for various indications. In recent years, several European HTA agencies have increased their literature review on TAVI, extending the assessment to TF TAVI for patients at low surgical risk. HTAs may arrive at different conclusions regarding the incremental therapeutic benefit of innovative medical devices, primarily due to differences in preference and interpretation of data, such as trial design, relevant endpoints, patient subgroups, treatment comparators, and other factors. This could mislead stakeholders and physicians as well as create divergence in national reimbursement status and accessibility. In addition, in presence of a frequent discrepancy among the conclusions achieved considering the evaluations by HTA agencies, the general robustness of the assessing system might be compromised. TAVI technologies were subject to a high number of assessments in Europe and many other regions in the world. Although these assessments have been using approximately the same evidence published in the literature, the assessment methods and the interpretation of the outcomes, the recommendations, and the degree of use were somehow different.

Given what is stated in the introductory section, the objectives of the study were as follows:

- To analyze the heterogeneity in terms of assessment methods and interpretation of the outcomes deriving from different TAVI assessments related to low-risk (LR) patients group;
- To explore the limitations of current HTAs on TAVI and the potential impact on access to patients.

## 2. Methods

For the purpose of this analysis, a review of the literature based on a manual research was performed. A population, intervention, comparators, and outcome (PICO) model (Table 1) was used to gather HTA reports assessing TAVI in the treatment of patients affected by symptomatic severe aortic valve stenosis at low surgical risk (LR). Given that TAVI was approved and expanded for low-risk patients in 2019, a temporal limit starting from 2019 was considered in our search strategy to gather the latest evidence on this subgroup.

**Table 1:** PICO model.

Population	Patients with symptomatic severe aortic valve stenosis at low surgical risk
Intervention	TAVI
Comparator	SAVR
Outcomes	Efficacy Safety Economic impact

**Figure 1:**
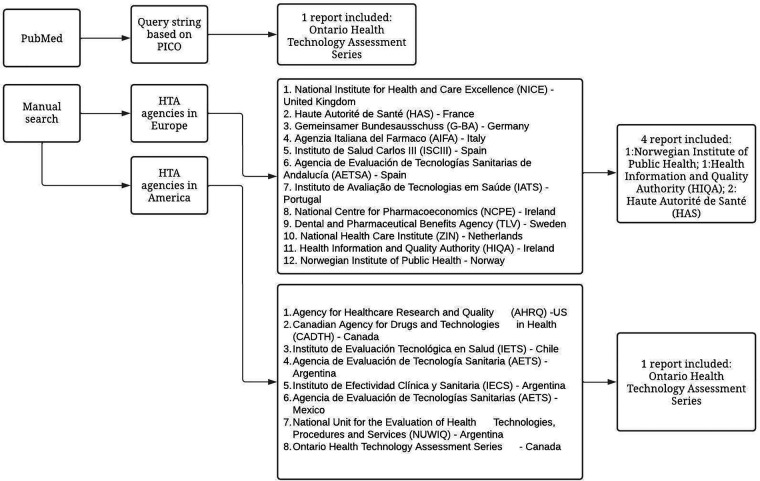
Study selection process.

In Table 1, we report the PICO utilized for the manual research while in Figure 1, we report the study selection process and the HTA agencies that were consulted during the research.

At the end of the study selection process, the following HTA reports that focused on LR population were included in the analysis:

• Health technology assessment of TAVI in patients with severe symptomatic aortic stenosis at low and intermediate risk of surgical complications (Health Information and Quality Authority, Ireland) (5).
• Transcatheter aortic valve implantation in patients with severe aortic valve stenosis at low surgical risk: a health technology assessment (Ontario Health Technology Assessment Series, Canada) (6).
• TAVI vs. SAVR for patients with severe aortic stenosis and low surgical risk and across surgical risk groups: a health technology assessment [Norwegian Institute of Public Health (NIPH), Norway] (7).

The authors discretionally chose to also include the Irish report even if it is not focused only on LR patients since the assessment reports relevant information for the heterogeneity analysis. Furthermore, during the manual search, we also included the assessments from the Haute Autorité de Santé (HAS). HAS recently published a series of reports developed by the National Commission for the Assessment of Medical Devices and Health Technologies (CNEDIMTS) on TAVI technologies (8–11). These reports, contrary to those mentioned above, do not strictly follow the EUnetHTA CoreModel and are specific assessment to a particular device. However, they were included in the heterogeneity analysis as they report relevant information to our study in terms of device safety and efficacy and in terms of potential economic impact. In this context, it should be noted that in the latest ESC guidelines, new scores have been developed to estimate the risk in patients undergoing TAVI, with better accuracy and discrimination than the surgical risk scores (1). However, since the research was started in July 2021, the HTAs that were included in this manuscript still reported surgical risk as one of the criteria for identifying the target population, and in this case, we focused on those reports that reported clinical information or economic implications of TAVI in low-risk patients. Thus, three HTA reports (5–7) and two HAS assessments (8, 9) were identified and included in the heterogeneity analysis. Our review focused first on reporting the main recommendations of the identified assessments and analyzed afterwards the type of evidence used to evaluate the efficacy and safety of TAVIs in LR patients (randomized clinical trials, guidelines, meta-analyses, registers, etc.). Then, the results of the assessments were also compared investigating the economic domain and assessing the type of model, the time horizon considered, the specific threshold used to determine the cost-effectiveness profile of the technology being assessed, the assumptions utilized, the type of device used to populate the economic evaluations, the type of sensitivity analysis performed, and the main results achieved. In Tables 2, 3, we summarize both for clinical and economic domains the characteristics of the assessments included, reporting the PICO for each assessment. In the Supplementary Tables S1, S2, we report an overview of the HTA reports included for the efficacy, safety, and economic domains. Finally in the Supplementary Appendix, we report the main characteristics of the RCTs on the LR population included in the HTA evaluations considered within the study.

**Table 2:** Characteristics of the clinical assessments included.

	**Clinical domains**				
	**Norway**	**Canada**	**Ireland**	**France (SAPIEN 3)**	**France (CoreValve)**
Population	Patients with severe aortic stenosis across all surgical risks	Adults with severe aortic valve stenosis and specific low surgical risk	Patients with aortic stenosis at low or intermediate surgical risk	Patients with symptomatic severe native aortic stenosis at low surgical risk	Patients aged at least 70 years at low risk (STS < 4%) with symptomatic severe native aortic stenosis
Intervention	Catheter-based implantation of aortic valves (TAVI)	Transcatheter aortic valve implantation	TAVI as a therapeutic intervention for the defined target population	SAPIEN 3	CoreValve Evolut R/PRO
Comparator	Open surgery aortic valve replacement (SAVR)	Surgical aortic valve replacement with a bioprosthetic valve	SAVR	For patients with contraindication to aortic valve replacement surgery or operable patients with an STS score ≥ 4%: implanted aortic valve bioprostheses For operable patients with an STS score < 4%: aortic valve replacement surgery	Aortic valve replacement surgery
Outcome	• Mortality at 30 days or longest available (all-cause mortality, cardiovascular mortality, non-cardiovascular mortality) • Improvement of symptoms [reduction in New York Heart Association (NYHA) class] • Improvement of indicators for health-related life quality (e.g., EQ-5D score, SF-12 score, KCCQ score) • Procedural success (successful implantation) • Hemodynamic function of aortic valve • Days in ICU (ICU stay) • Days in hospital • Readmission to the hospital due to heart attack • Need for permanent pacemaker implantation • All undesired outcomes (e.g., vascular complications, stroke, transient ischemic attack (TIA), major bleeding, re-intervention, heart attack ≤72 h after procedure, new or worsened atrial fibrillation-flutter, moderate or severe valve leakage (regurgitation), acute kidney damage, radiation damage patient or staff	• Composite endpoint (all-cause mortality, stroke, or rehospitalization at 1 year; all-cause mortality or disabling stroke at 2 years) • All-cause mortality • Stroke and transient ischemic attack • Aortic valve re-intervention • Rehospitalization • Change in New York Heart Association (NYHA) scores • Quality of life • 6 min walk test • Valve hemodynamics (aortic valve area and aortic valve gradient) • Length of hospital stay for TAVI and SAVR procedures • Procedural complications • Life-threatening or disabling bleeding • Major vascular complications • Acute kidney injury • New-onset atrial fibrillation • Myocardial infarction • New permanent pacemaker implantation • New left bundle branch block • Moderate-to-severe paravalvular aortic regurgitation • Valve thrombosis • Leaflet thickening and leaflet mobility restriction	Clinical efficacy outcomes: • Mortality • Symptom improvement • Health-related quality of life • Health service utilization Safety outcomes: • Any major or minor adverse event • Radiation causing harm to both patient and staff	Evaluation of the therapeutic effect/adverse effects, risks related to the use	Evaluation of the therapeutic effect/adverse effects, risks related to the use
Study design	Systematic reviews and meta-analyses	• Randomized controlled trials • Health technology assessments, systematic reviews, and meta-analyses of RCTs if they included the most recent RCTs in patients at low surgical risk	Clinical efficacy - Randomized controlled trials Safety - Randomized controlled trials - Real-world data derived from published studies from prospective national registries	HTA report (ONTARIO), systematic reviews and meta-analyses, randomized controlled trials and registries	HTA report (HIQA), systematic reviews, meta-analyses, randomized controlled trials

**Table 3:** Characteristics of the economic assessments included.

	Economic domains				
	Norway	Canada	Ireland	(HAS SAPIEN 3)	(HAS CoreValve)
Population	Patient with severe aortic valve stenosis and low surgical risk	Adults with severe aortic valve stenosis and low surgical risk	Patients with aortic stenosis at low or intermediate risk of surgical complications	Symptomatic severe aortic stenosis at low surgical risk	Low-risk surgical patients treated for severe, symptomatic aortic stenosis
Intervention	TAVI	Transcatheter aortic valve implantation	TAVI/transcatheter aortic valve replacement (TAVR)	SAPIEN 3	CoreValve Evolut R/Evolut PRO
Comparator	Aortic valve replacement with conventional surgery (SAVR)	Surgical aortic valve replacement	SAVR	Aortic valve replacement with conventional surgery (SAVR)	SAVR
Outcome	• Costs • Stroke • Acute kidney injury • Myocardial infarction • Major vascular complications • New pacemaker implantation • Life-threatening bleeding • Paravalvular regurgitation • New-onset atrial fibrillation	• Costs • Health outcomes (e.g., QALYs) • Incremental costs • Incremental effectiveness • ICER	Any measure of costs and benefits	Incremental cost-effectiveness ratio Budget impact	Evaluate the safety and efficacy of Medtronic TAVI for surgical aortic valve replacement for the treatment of patients with severe aortic stenosis with a low predictive risk of surgical mortality at 30 days
Study design	Cost-utility analyses	Cost-benefit analyses, cost-effectiveness analyses, or cost-utility analyses	Economic evaluations	Cost-utility analysis and budget impact	Cost-utility analysis and budget impact

## 3. Results

### 3.1. Safety and efficacy domains

*Health technology assessment of transcatheter aortic valve implantation (TAVI) in patients with severe symptomatic aortic stenosis at low and intermediate risk of surgical complications—Health Information and Quality Authority (HIQA)—2019* (5)

#### 3.1.1. Objective

The objective of this HTA was to assess the utilization of TAVI as a therapeutic approach for patients in Ireland with severe aortic stenosis who are at low or intermediate risk for surgical complications. A systematic review was conducted to identify pertinent studies on the application of TAVI in the treatment of patients with severe symptomatic aortic stenosis at low and intermediate surgical risk.

#### 3.1.2. Data source

The efficacy data primarily relied on non-inferiority trials with short-term follow-up, involving second- and third-generation TAVI devices. The trials included in this assessment were international, randomized, and multicenter clinical trials, as well as real-world data from prospective national registries, such as the STACCATO trial in Denmark, the placement of aortic transcatheter valves (PARTNER) 2 trial in the USA and Canada, and the NOTION trial in Denmark and Sweden. The remaining three trials, Evolut Low Risk, PARTNER 3, and surgical replacement and transcatheter aortic valve implantation (SURTAVI), were larger multinational trials with a focus on the USA and Canada and additional centers in Japan, Australia, New Zealand, and Europe. All the included trials were designed to demonstrate non-inferiority, with some having the potential to show superiority (e.g., PARTNER 3 for the low-risk population). However, due to important design differences and potential variation in adverse event profiles between generations of valves and manufacturers, the limited trial and registry data available restrict the ability to conduct a detailed analysis. In addition, the iterative development of devices and contemporary changes in the management of patients undergoing aortic valve replacement (AVR) may limit the applicability of earlier trial data on TAVI. The majority of the evidence for patients who are at intermediate or mixed low-to-intermediate surgical risk is derived from the studies involving second-generation TAVI devices while for patients at low surgical risk, the evidence was based on second- and third-generation TAVI devices. In this context, it is also relevant to underline that the clinical and safety outcomes can be influenced even by subtle changes to the devices. Most of the trials focused on a specific device from one manufacturer, which may also limit the generalizability of the findings to real-world settings where clinicians may choose the most appropriate device based on the clinical context of the patient. However, the availability of registry data that are not device-specific supports the overall finding that TAVI devices are non-inferior in terms of main clinical effectiveness outcomes.

#### 3.1.3. Recommendation

Therefore, based on the available evidence, the advice of HIQA to the Minister for Health, the Department of Health, and the Health Service Executive (HSE) was that TAVI should be made available for patients aged 70 years and older with severe symptomatic aortic stenosis at low and intermediate surgical risk within the Irish public healthcare system.

*Ontario health technology assessment series transcatheter aortic valve implantation in patients with severe aortic valve stenosis at low surgical risk: a health technology assessment—2020 (6)*

#### 3.1.4. Objective

The aim of this HTA was to evaluate the evidence for TAVI as a treatment strategy for Canadian patients with severe aortic stenosis who are at low risk of surgical complications.

#### 3.1.5. Data source

Two RCTs comparing TAVI and SAVR in patients at low surgical risk, namely, the PARTNER 3 and the Evolut LRT studies, were identified and included in the analysis. The results from PARTNER 3 showed that TAVI significantly improved the composite endpoint of all-cause mortality, stroke, or rehospitalization compared with SAVR at 1 year. The Evolut study demonstrated that TAVI was non-inferior to SAVR in terms of the composite endpoint of all-cause mortality and disabling stroke at 2 years. However, longer follow-up is needed to better understand the durability of TAVI valves and draw definitive conclusions on the long-term effects of TAVI compared with SAVR beyond 1 year in patients with severe aortic valve stenosis and low surgical risk, as stated by the study authors. Moreover, patients and caregivers perceived that TAVI resulted in less pain and shorter recovery time compared with SAVR, with most patients returning to their usual activities more quickly.

#### 3.1.6. Recommendation

Based on the evidence reviewed in this report, Ontario Health, guided by the Ontario Health Technology Advisory Committee, recommends public funding for transcatheter aortic valve implantation in adults with severe aortic valve stenosis who are at low surgical risk.

*Health technology assessment: transcatheter aortic valve implantation (TAVI) vs. surgical aortic valve replacement (SAVR) for patients with severe aortic stenosis and low surgical risk and across surgical risk groups—Norwegian Institute of Public Health—2021* (7)

#### 3.1.7. Objective

The aim of this HTA was to evaluate the evidence for TAVI as a treatment strategy for patients in Norway with severe aortic stenosis, both at low surgical risk and across different surgical risk groups.

#### 3.1.8. Data source

The search strategy included 15 systematic reviews published since April 2019, which assessed the effects of TAVI/TAVR compared with SAVR in patients with severe aortic stenosis. Among these reviews, 11 summarized studies on low-risk patients, two assessed patients at low-to-intermediate surgical risk, and the last two considered patients across all surgical risk groups. The systematic reviews included the two most recently published RCTs on low-risk patients, namely, PARTNER 3 and Evolut LRT. Although most reviews reported the methods for analyzing results and conducting meta-analyses, only three of them used the grading of recommendations, assessment, development, and evaluation (GRADE) to assess the certainty of the effect estimates by evaluating the risk of bias. Some of the reviews were downgraded in terms of quality due to lack of risk of bias assessment in the included primary studies or lack of reporting on risk of bias assessment. The authors chose to report only the results of a high-quality Cochrane systematic review with GRADE assessment by Kolkailah et al. (12) updated in April 2019, which included four RCTs and a total of 2,818 randomly assigned patients to TAVI (*n* = 1,416) or SAVR (*n* = 1,402). The two most recent trials, EVOLUT (2019) (*n* = 1,468) and PARTNER 3 (2019) (*n* = 1,000), had the largest number of patients, and the other two studies included were NOTION (2015) (*n* = 280) and STACCATO (2012) (*n* = 70). The authors concluded that despite the short-term non-inferior outcomes, more data and long-term follow-up are needed to further assess and validate the outcomes, particularly in the low surgical risk population.

#### 3.1.9. Recommendation

In addition to the report from the NIPH, the Decision Forum of Regional Health Authorities (RHAs) published a decision stating that catheter-based implantation of aortic valves can be used in the treatment of patients with severe aortic stenosis in hospitals that are already performing cardiac surgery, across all risk groups.

*Haute Autorité de Santé (HAS)—National Commission for the Evaluation of Medical Devices and Health Technologies* (8) *(SAPIEN 3)*

#### 3.1.10. Objective

This assessment was a renewal one for all indications and is not specific to low-risk patients. However, the authors included evidence also for this subgroup. The commission focused on patients with severe symptomatic native aortic stenosis (SVAoi < 0.5 cm^2^/m^2^). For operable patients with an Society of Thoracic Surgeons (STS) score of <4%, in the French healthcare setting, the indication is limited to patients over 65, with a tricuspid orifice, without indication for mitral or coronary valve surgery (common trunk and/or SYNTAX > 32), and associated with an anatomy favorable to the transfemoral approach.

#### 3.1.11. Data source

The following data were retained:

Non-specific data:

• A technology assessment report from the Canadian agency ONTARIO on the management of TAVI in intermediate-risk patients.
• A meta-analysis of observational studies (*n* = 15) comparing efficacy and safety data from 4,496 patients who benefited from the installation of an EDWARDS SAPIEN XT prosthesis or EDWARDS SAPIEN 3.
• The PARTNER IIA randomized controlled study at 5 years of follow-up to compare the EDWARDS SAPIEN XT prosthesis with the conventional aortic valve replacement surgery in more than 2,000 patients at intermediate risk.
• The final 5-year follow-up study report from the French registry France 2 covering 4,153 patients.

Specific data:

• The PARTNER II S3i study on the quality-of-life component at 1 year of follow-up in nearly 3,000 patients at intermediate risk having benefited from the installation of an EDWARDS SAPIEN 3 valve, EDWARDS SAPIEN XT, or a conventional surgical procedure.
• The updated results of the PARTNER 3 study at 2 years of follow-up in low-risk patients to compare the valve EDWARDS SAPIEN 3 with conventional surgical aortic valve replacement.

#### 3.1.12. Recommendation

The Commission will also take note of long-term data term (10 years) of the France-TAVI registry. The National Commission for the Evaluation of Medical Devices and Health Technologies considered that the evidence available on TAVI in LR is sufficient for the renewal of registration and modification of the conditions of registration on the list of Products and Services provided for in Article L.165-1 of the Social Security Code. The indication must be made of a multidisciplinary meeting taking into account the risk scores and the associated comorbidities. For operable patients with an STS score of <4%, the indication is limited to patients over 65 years of age, with a tricuspid orifice, without indication for mitral or coronary valve surgery (common trunk and/or SYNTAX > 32), and associated with an anatomy favorable to the transfemoral approach. Patients with a life expectancy of less than 1 year considering extracardiac factors (comorbidities) or with significant calcifications in the subaortic outflow chamber are not eligible for the technique.

*Haute Autorité de Santé (HAS)—National Commission for the Evaluation of Medical Devices and Health Technologies* (9) *(CoreValve)*

#### 3.1.13. Objective

The commission focused on patients aged at least 70 years who are at low risk (STS < 4%) with symptomatic severe native aortic stenosis (SVAoi < 0.5 cm^2^/m^2^) over the tricuspid orifice, without indication for mitral or coronary valve surgery (common trunk and/or SYNTAX > 32), and associated with an anatomy favorable to the transfemoral route.

#### 3.1.14. Data source

The following data were selected:

Non-specific data:

• An Irish HIQA technology appraisal report on the management of TAVI in intermediate- and low-risk patients.
• A non-specific meta-analysis to compare percutaneous vs. surgical aortic valve replacement in low-risk patients (STS < 4%). This meta-analysis included four randomized controlled trials involving 2,887 patients followed up for at least 1 year.
• A prospective, multicenter, controlled, randomized study at a 5-year follow-up to compare the efficacy and safety results of the CoreValve range with conventional surgery in 280 all-comers patients aged at least 70 years.

Specific data:

• A prospective, multicenter, controlled, randomized study to evaluate the safety and efficacy of transcatheter implantation of the CoreValve line of bioprostheses in patients with severe symptomatic or non-symptomatic aortic stenosis at low surgical risk for surgical aortic valve replacement. The primary endpoint was a composite of all-cause death and disabling stroke up to 2 years of follow-up. This endpoint was tested in non-inferiority in 850 patients who reached a 1-year follow-up (protocol-specified interim analysis).

#### 3.1.15. Recommendation

In conclusion, the National Commission for the Evaluation of Medical Devices and Health Technologies considers that the expected service is sufficient for the modification of the conditions of registration on the list of products and services provided for in article L.165-1 of the social security code. The Commission retains the following indications: patients aged at least 70 years at low risk (STS <4%) with severe symptomatic native aortic stenosis (SVAoi < 0.5 cm^2^/m^2^) on the tricuspid orifice, without indication for mitral or coronary valve surgery (common trunk and/or SYNTAX > 32), and associated with an anatomy favorable to the transfemoral route. The indication should be determined in a multidisciplinary meeting taking into account risk scores and associated comorbidities. Patients with a life expectancy of less than 1 year due to extracardiac factors (comorbidities) or with significant calcifications in the subaortic outflow tract are not eligible for the technique (non-indication).

#### 3.1.16. Safety and efficacy: summary

In the previous sections, therefore, the main results and the type of evidence included from the evaluations of the various HTA agencies are reported. A summary of the results just described is presented in Supplementary Table S1. The table reports the following elements:

• Inclusion criteria of the target population.
• Device.
• Study included.
• Inclusion criteria of the evidence included.
• STS score.
• Safety and efficacy (conclusions).
• Primary objective.
• Clinical guidelines included.
• Recommendations.

### 3.2. Analysis of the heterogeneity: safety and efficacy domains

Despite that the results on TAVI expressed in the HTAs are all quite positive, as can be seen in Supplementary Table S1, there is a certain degree of heterogeneity in the evidence factored and in the recommendations on TAVI. This section provides an overview of the main heterogeneity elements found in the analysis of the HTA reports and in the HAS assessments. We found that the reasons for this disparity lied mainly on the following elements: the main driver of disparity derives from the type of evidence considered (systematic reviews, real-world registries, clinical trials, meta-analysis). Regarding low-risk subgroup, the Canadian and the Irish report considers the PARTNER 3 and Evolut LRT randomized clinical trials, while the Norwegian report considers the STACCATO and NOTION studies in addition to the two just mentioned. The SAPIEN 3 HAS assessment takes into account the HTA report from Ontario and the PARTNER S3i/PARTNER 3 trials, while the CoreValve HAS assessment takes into account the HIQA report and the Evolut LRT trial; however, non-specific evidence such as meta-analyses or literature reviews are also considered in HAS reports. Moreover, the search strategy of the Norwegian report includes 11 reviews summarizing the evidence on low-risk patients. This poses a limitation in the evaluation since the evidence considered is characterized by major design differences that make it difficult to assess differences in the safety and efficacy profile of different TAVI technologies. Moreover, most of the reviews included in the HTA reports did not report assessment of risk of bias in included primary studies. Therefore, the inclusion and exclusion criteria of the studies are different and characterized by heterogeneity in the evaluation. Thus, there are several sources of heterogeneity in the evaluation of TAVI. One such source is the variability in the generation of devices used in the studies. As devices undergo iterative development and contemporary changes occur in the management of patients undergoing aortic valve replacement, recommendations on TAVI may have limited generalizability. Most of the trials analyzed in the HTA reports included in the evaluation used a specific device from one manufacturer, which may restrict the applicability of the findings to real-world settings and limit the clarity of decision-making for policymakers in the specific evaluation setting. Another element of heterogeneity is the use of the GRADE methodology to evaluate the quality of clinical evidence included in the reports. The Norwegian report appears to be the only one that utilized this methodology to assess the evidence for inclusion in the assessment. Furthermore, some HTA reports did not investigate the reasons why certain meta-analyses found statistically significant differences in important outcomes, such as mortality, in favor of TAVI, while others based on similar studies did not reach the same conclusions.

### 3.3. Results: economic domain

*Health technology assessment of transcatheter aortic valve implantation (TAVI) in patients with severe symptomatic aortic stenosis at low and intermediate risk of surgical complications—Health Information and Quality Authority (HIQA)—2019* (5)

#### 3.3.1. Cost-utility analysis

As there was no appropriate economic model available for Ireland, a specific probabilistic Markov model was created to evaluate the cost-effectiveness and budgetary implications of TAVI vs. SAVR for patients with severe symptomatic aortic stenosis who are at low and intermediate risk of surgical complications. The analysis was conducted from the perspective of the publicly funded health and social care system. For the cost-utility analysis (CUA), costs and outcomes were simulated over a 15-year time horizon, which was assumed to be the lifespan of a TAVI valve. Future costs and outcomes were discounted at 4% per annum, and the results were presented using a conservative willingness-to-pay threshold of €20,000 per quality-adjusted life year (QALY) gained. The model used existing evidence on currently used TAVI devices to assess the clinical effectiveness of the procedure compared with SAVR. Specifically, data from the PARTNER 2 trial on the second-generation SAPIEN XT valve were used for intermediate-risk patients, and data from the PARTNER 3 trial on the third-generation SAPIEN 3 valve were used for low-risk patients in the base case analysis. The results showed that TAVI was both less costly and resulted in a greater number of QALYs compared with SAVR in both intermediate and low surgical risk populations. In addition, a systematic review was conducted to evaluate the available evidence on the cost-effectiveness of TAVI vs. SAVR in low- or intermediate-risk patients with severe symptomatic aortic stenosis. However, the review identified seven studies, none of which were conducted in Ireland, and raised concerns about the quality and credibility of the economic evaluations, particularly in terms of model structure and input parameters. As a result, the evidence base was considered insufficient to determine the cost-effectiveness of TAVI for low or intermediate-risk patients in Ireland. Consequently, the economic model was developed specifically tailored to the Irish healthcare setting to address the question of cost-effectiveness and budget impact. While the model indicated that TAVI was cost-effective in intermediate- and low-risk patient populations, some uncertainty was observed, particularly in relation to costs. The cost-effectiveness of TAVI was found to be largely influenced by variations in the cost of the procedure, with a significant proportion of the estimated cost being influenced by the cost of the TAVI valve, which varies by manufacturer and can range substantially in price. Depending on the choice of valve, the cost-effectiveness of TAVI compared with SAVR may be positively or adversely impacted.

#### 3.3.2. Budget impact analysis

The budget impact analysis also took into account the impact of an ageing population on the demand for AVR. An increase in the proportion of the population aged 70 years or older will result in an increased demand for AVR, and consequently, a budget impact of TAVI and SAVR. Although the incremental cost of providing TAVI relative to SAVR is expected to remain budget neutral, the impact of an ageing population on the healthcare budget is a significant consideration. In the base case analysis, it was assumed that TAVI would be extended to patients at intermediate and low surgical risk. Over the first 5 years, TAVI was estimated to save €0.1 million (95% CI: €3.1–€2.9) compared with SAVR, which may be considered budget neutral. The estimated budget impact was found to be sensitive to changes in the cost of SAVR and TAVI procedures. However, if the additional procedures can be performed without requiring additional infrastructure, the estimated cost saving over 5 years is €0.8 (95% CI: €3.8–€2.3). Thus, the growing demand for AVR due to an aging population in Ireland will have a significant budget impact on TAVI and SAVR. However, the cost of implementing a TAVI pathway relative to SAVR is expected to remain budget neutral. The advantage of TAVI over SAVR is its efficiency, as it is associated with a shorter hospital stay. Therefore, expanding the TAVI care pathway to include patients with severe symptomatic aortic stenosis at low and intermediate surgical risk should be considered in the Irish public healthcare system. Current clinical evidence suggests that TAVI is as effective as SAVR in terms of cardiac and all-cause mortality. In addition, TAVI has the benefit of shorter hospital stays and improved health-related quality of life in the short-term due to its less invasive nature. TAVI is also considered a highly cost-effective treatment option for patients aged 70 years and older at low or intermediate surgical risk, when compared with SAVR. The estimated budget impact over 5 years of extending the TAVI care pathway to include approximately 100 patients at low and intermediate surgical risk is expected to be budget neutral, even when accounting for the cost of additional catheterization laboratory capacity. Furthermore, increased utilization of TAVI as an alternative to SAVR is anticipated to result in reduced hospital stays, decreased demand for ICU beds and theater time, and potentially freeing up resources to address other demands within the healthcare system.

*Ontario health technology assessment series transcatheter aortic valve implantation in patients with severe aortic valve stenosis at low surgical risk: a health technology assessment—2020* (6)

#### 3.3.3. Cost-utility analysis

The economic review conducted in this report identified a study that compared the cost-effectiveness of TAVI with SAVR specifically in low-risk patients with severe aortic valve stenosis. The study found that “*TAVI was more effective in terms of generating quality-adjusted life-years (QALYs), but it was also more expensive. The costs associated with TAVI devices were significantly higher (around $25,000) compared to SAVR (around $6,000). A cost-effectiveness analysis, conducted from the perspective of the Ontario Ministry of Health, revealed that TAVI was slightly more effective but also more expensive than SAVR, with incremental cost-effectiveness ratios (ICERs) of $27,196 per QALY and $59,641 per QALY for balloon-expandable and self-expanding TAVI, respectively. Furthermore, the study found that balloon-expandable TAVI was less costly on average (by $2,330) and slightly more effective (by 0.02 QALY) compared to self-expanding TAVI. Among the three interventions, balloon-expandable TAVI had the highest probability of being considered cost-effective, being the preferred option in 53% and 59% of model iterations at willingness-to-pay values of $50,000 and $100,000 per QALY, respectively. Self-expanding TAVI was preferred in less than 10% of iterations*” (6). However, there was no quantitative or qualitative evidence on patient preferences and values specific to the low-risk surgical group. Patients and caregivers of patients with severe aortic valve stenosis at low surgical risk perceived that TAVI minimized pain and recovery time, and most patients who had TAVI were able to return to their usual activities more quickly than if they had undergone SAVR. Direct patient and caregiver consultations indicated a preference for TAVI over SAVR.

#### 3.3.4. Budget impact analysis

The estimated 5-year budget impact of publicly funding TAVI in adults at low surgical risk, compared with the current scenario where SAVR is used, was between $37 and $45 million yearly ($21–$43 million of this would be for TAVI) in the new scenario where there is a mix of TAVI and SAVR. This would result in an additional annual budget impact of $5–$8 million per year, considering the current spending on SAVR. However, these results should be interpreted with caution due to limitations. First, considering the existing infrastructure, it was assumed that TAVI would be rapidly adopted by 50% of eligible patients in the first year. However, several factors could influence the uptake rate of TAVI, such as system and infrastructure readiness, funding allocation, or backlogs of patients at high surgical risk who are already funded for TAVI, which could either increase or decrease the uptake rate. Second, estimating the proportion of patients at low surgical risk and identifying the number of patients who would be eligible or most likely to receive TAVI were challenging. While the determination of risk and eligibility takes into account factors such as the STS score and age, it ultimately depends on the decision of a heart team, which considers multiple risk factors and comorbidities. Variations in these proportions could result in a budget impact ranging from an additional $3–$6 million annually to $6–$11 million annually. Third, the authors did not account for potential revision surgeries in their relatively short time horizon. When TAVI is used in younger patients, there may be a higher likelihood of revision surgeries, which could impact costs and outcomes. Finally, the long-term durability of TAVI is still uncertain. As more evidence becomes available, the durability of TAVI should be considered in future analyses.

*Health technology assessment: transcatheter aortic valve implantation (TAVI)* vs. *surgical aortic valve replacement (SAVR) for patients with severe aortic stenosis and low surgical risk and across surgical risk groups—Norwegian Institute of Public Health—2021* (7)

#### 3.3.5. Cost-utility analysis

In the economic evaluation, the authors of the report compared the cost-effectiveness of TAVI with SAVR for patients with severe aortic stenosis at low risk. They used clinical data from the multicenter trial PARTNER 3 to inform their analyses. The base case scenario in their cost-effectiveness analysis showed that the “*total expected average intervention-related costs per patient over a 15-year period were approximately 428,000 NOK for SAVR and 393,000 NOK for TAVI, including costs of the procedures and treatment of complications. This made TAVI about 35,000 NOK less costly per patient over the time horizon considered, despite higher procedure costs (with a difference of about 31,000 NOK) used in the model. At 1 year, both procedures had similar total costs. In terms of effectiveness, TAVI patients accumulated slightly more QALYs (quality-adjusted life years) with a difference of about 0.055 QALYs*” (7). This made TAVI a dominant alternative, being better both in terms of effectiveness and less costly compared with SAVR in the base case analysis. However, these results should be interpreted with caution as sensitivity analyses showed that the procedure cost parameters had the most influence on the results. The model was based on data from a 1-year follow-up only, and long-term studies on survival, procedure-related complications, longevity of prostheses used in both TAVI and SAVR, and the need for future re-intervention are yet to be established and documented. The authors assumed no procedure-related complications beyond 1 year following the aortic procedure. There were considerable variations in clinical practice in Norway regarding the length of hospital stay among hospitals performing TAVI procedures ranging from 1 to 4 days in 2019. In summary, the cost-utility analysis found that TAVI was slightly more effective (with an incremental effectiveness of 0.05 QALYs) and less costly (with a savings of 35,000 NOK) compared with SAVR for patients at low surgical risk. However, it should be noted that the analysis was based on 1-year follow-up data from the PARTNER 3 study, and the long-term mortality and adverse events for both TAVI and SAVR beyond this period are still uncertain. The results were also sensitive to variations in procedure costs.

#### 3.3.6. Budget impact analysis

The budget impact analysis indicated that the introduction of TAVI for low-risk patients is likely to be cost-neutral in the short-term, but the costs of capacity expansion were not taken into account. The calculated absolute shortfall for patients with severe aortic stenosis and low surgical risk, compared with the general population, was estimated to be two QALYs.

*Haute Autorité de Santé (HAS)—National Commission for the Evaluation of Medical Devices and Health Technologies* (8) *(SAPIEN 3)*

#### 3.3.7. Cost-utility analysis

The objective of the economic evaluation presented in the HAS report was to assess the cost-effectiveness profile of SAPIEN 3 in the treatment of severe symptomatic aortic stenosis in patients with low surgical risk in France. The baseline analysis, based on quality-adjusted life years gained, is invalidated by the major caveat regarding the sources of the quality-of-life data and the method of estimating the utilities associated with the health states in the model. Nevertheless, the economic analysis in terms of life years gained is acceptable. At the claimed price of €17,175.40 including VAT, it results in the dominance of valve replacement surgery by SAPIEN 3. According to the proposed modeling, this strategy would save 4months of life and induce a cost reduction of €7,737 over a 15-year time horizon. However, it is subject to uncertainty due to the following factors:

- The modeling is based exclusively on health states defined according to the presence of a pre-selected serious adverse event (SAE) and does not include other important, severe, or serious events, independent of the events defining the states “atrial fibrillation” and “disabling stroke”;
- The lack of robust inclusion of CoreValve Evolut PRO/R, a competitor device to SAPIEN 3 in the indication evaluated, in the comparator scenario analysis. Indirect comparisons are limited in scope in the probabilistic sensitivity analyses performed (i.e., very wide credibility intervals for all-cause mortality, atrial fibrillation, and disabling stroke);
- The use of relative mortality risks from non-SAPIEN 3 specific studies and extrapolation assumptions based exclusively on the opinion of the Scientific Committee established for this economic analysis.

#### 3.3.8. Budget impact analysis

Regarding the budget impact, at the claimed price of €17,175.40, the introduction of SAPIEN 3 results in a reduction in health insurance expenditure of around €67 million over 5 years. This estimate is subject to a high degree of uncertainty and is therefore difficult to use due to the following:

- The failure to take into account other competing schemes (e.g., CoreValve Evolut PRO/R) in the baseline analysis. As a result, the budgetary impact of the introduction of SAPIEN 3 is estimated with a high degree of uncertainty about the amount of savings generated by the introduction of SAPIEN 3, which does not take into account the contribution of the other competing devices to health insurance expenditure;
- Limitations related to the consideration and valuation of integrated costs: on the one hand, the limitations of the efficiency analysis (e.g., absence of consideration of the costs of serious or severe adverse events, independent of the events defining the health states of the efficiency model) and on the other hand, the perspective of long-term complications, which is not limited to the perspective of compulsory health insurance.

Moreover, the scenario analyses integrating CoreValve Evolut PRO/R do not allow to explore the uncertainty on the estimation of the budgetary impact related to the introduction of SAPIEN 3. Indeed,

- The scenario analyses including CoreValve Evolut PRO/R result in an estimated budgetary impact related to the introduction of TAVI, without distinguishing the shares related to SAPIEN 3 and CoreValve Evolut PRO/R;
- The structure of the budget impact model for this scenario analysis is different from that of the baseline analysis (i.e., the Markov traces on which the two models are based are not similar). This makes it impossible to compare the budget impact results obtained in the baseline analysis and the scenario analysis.

*Haute Autorité de Santé (HAS)—National Commission for the Evaluation of Medical Devices and Health Technologies* (9) *(CoreValve)*

#### 3.3.9. Cost-utility analysis

The objective of the economic evaluation presented in the HAS report was to assess the cost-effectiveness profile of CoreValve Evolut R/PRO in the treatment strategy compared with surgical aortic valve replacement. This evaluation is being conducted in the context of applications to change the conditions of listing for CoreValve Evolut R/PRO to extend the indication to patients with low surgical risk.

Over a time horizon of 15 years, at the price claimed by the manufacturer of €15,419.21 including tax, the analysis of CoreValve Evolut R/PRO for the management of patients with severe aortic stenosis at low surgical risk resulted in the following:

- A total cost increment of €708.15 per patient for the CoreValve Evolut R/PRO strategy and health benefits of 0.09 and 0.12 for adjusted life years gained and quality-adjusted life years gained for the CoreValve Evolut R/PRO vs. aortic valve replacement surgery;
- An ICER of €7,571.39/ALY and €5,893.01/QALY vs. aortic valve replacement surgery.

The main factor leading to very high variability in ICER is the stroke rate observed in the clinical trial for the surgery strategy. For the probabilistic sensitivity analyses, the willingness-to-pay (WTP) for which CoreValve Evolut R/PRO has an 80% probability of being cost-effective compared with surgery is €80,000/QALY. The uncertainty related to the impact of the loss of quality of life due to stroke is partly unquantifiable due to the following:

• Limitations of the available data on quality of life in the Evolut Low Risk trial on stroke and questions about the robustness of the estimate of this loss of utility extracted from the literature (i.e., method of valuing utilities and comparability populations);
• The simplified structure of the model (lack of description of the post-stroke state as a function of the evolution of the health status of the patient and the occurrence of long-term intercurrent events).

#### 3.3.10. Budget impact analysis

Regarding the budget impact model at the claimed price of €15,419.21 including tax, the result on health insurance expenditure deriving from the introduction of TAVI in the market (including CoreValve Evolut R/PRO but also other TAVI) in patients with severe symptomatic native aortic valve stenosis who are at low risk for aortic valve replacement (STS score <4%) is €63.679.321 over a 5-year time horizon. The methodology used to assess the budgetary impact of CoreValve Evolut R/PRO is acceptable. However, the proposed analysis raises two major and one minor reservation. The two major reservations relate to the following:

- The estimation of market shares: the period over which the uptakes data were collected to define market shares and their evolution, the patients concerned by TAVI over this period (patients contraindicated for TAVI);
- The period (patients contraindicated to surgery) and the absence of information on the distribution of market shares between TAVIs (other than CoreValve Evolut R/PRO and SAPIEN 3) included in the model. The lack of information on the market share of TAVIs (other than CoreValve Evolut R/PRO and SAPIEN 3) integrated into the model from 2023 onward leads to a high degree of uncertainty in this estimate.
- Cost estimation: the robustness of the cost estimate of competing TAVIs is questionable. Indeed, the costs estimated for CoreValve Evolut R/PRO are applied to the whole of other TAVIs with the exception of the acquisition cost that is based on the published price of the Edwards Lifesciences SAPIEN 3 valve from Edwards Lifesciences.

#### 3.3.11. Economic domains: summary

A summary of the results just described is presented in Supplementary Table S2. The table reports the following elements:

• Type of model.
• Study design.
• Perspective.
• Time horizon.
• WTP threshold.
• Device type.
• Discount rate.
• Main assumption.
• Results.
• Sensitivity analysis.
• Data used.
• Conclusion.

### 3.4. Analysis of heterogeneity: economic evaluations

The economic evaluations assessed reported a time horizon of 15 years for cost-utility analyses and 5 years for budget impact models. Each HTA report or HAS assessment used the perspective of the national health system considering only direct healthcare costs. Despite some elements of heterogeneity in the selection of the sources to populate the models, the economic assessments conducted on the low-risk population of the included reports demonstrate a favorable or dominant cost-effectiveness profile for TAVI compared with SAVR. The first element of heterogeneity concerns the thresholds for assessing cost-effectiveness. However, this element is justified by the fact that in various contexts, there is no official acceptability threshold for health technologies. The second and most important element of heterogeneity is found in the type of device being analyzed. In fact, in the Canadian report, the analyses are carried out taking into account both the balloon-expandable and the self-expandable TAVI, while in the Norwegian, Irish, and French reports, the analyses are conducted on the SAPIEN 3. As mentioned in the methods section, two HAS evaluation reports were also included in the analysis. These reports being device-specific presented the economic evaluation analyses on SAPIEN 3 and CoreValve Evolut R.

Furthermore, as can be seen from Supplementary Table S2, there are also disparities in the data sources, the type of evidence, and the device generation used to populate the models. Moreover, each economic model used different assumptions although deterministic and probabilistic sensitivity analyses were performed in all the assessments to characterize the uncertainty around the parameters of the models. Specifically, the included HTA reports use different health states within Markovian models to describe the natural history of the condition. The Canadian report reports a Markov model with four health states (alive/well, disabling strokes, moderate paravalvular leak, and death). Conversely, the Norwegian report considers only three: alive and well, post major complications, and death. The more complex structure is reported by the cost-utility model developed in the Irish context that considers the following health states: alive/well, major complications (acute kidney injury, disabling stroke, myocardial infarction), post major complications, hospitalizations, and death. Even in the included French reports, there is a difference in terms of the health states used. The specific report on SAPIEN 3 considers four health states (alive and well, disabling strokes, treated atrial fibrillation, and death) whereas the specific report on CoreValve considers also four but different ones (no stroke, stroke, post-stroke, and death). In addition to this, the survival estimates used in the cost-utility models are different and often based on the general population. Since the trials provided data on all-cause mortality up to 1 year in low-risk patients and 2 years in intermediate-risk patients, the Irish report utilized the National Life Tables for Ireland from 2015, stratified by age and sex, to estimate all-cause mortality beyond these time points. As patients with severe symptomatic aortic stenosis are believed to be at a higher risk of mortality compared with the general population, a higher relative risk was applied to the all-cause mortality rates using data from Chakos et al. (13). In the Norwegian report, data for mortality in the acute phase at 30 days were directly applied in the model, since trial data beyond 1 year were not available during the analysis. For this period, extrapolation was performed by assuming that all patients with aortic stenosis following aortic valve replacement have an increased risk of death compared with the general population. For patients who were alive and well, age-adjusted mortality data for the general Norwegian population, recalculated to monthly probabilities and multiplied by different hazard ratios depending on health status, were used for extrapolation. In conclusion, despite the heterogeneity of the aforementioned elements and the different structures of the Markov models, the results of economic evaluations in different contexts report either a cost-effectiveness profile or a dominance profile. To our knowledge, currently the only negative recommendation in terms of cost-effectiveness of TAVI in LR population came from the National Institute of Healthcare Excellence (NICE). NICE on the latest HVD guidelines (14) did not have concern on the safety or efficacy profile of TAVI in the low-risk population, but in their assessment, the technology was not cost-effective considering the acceptability threshold in the UK setting. Although the NICE report was not examined in depth as it is not specific to low-risk patients, it is worth emphasizing that the results achieved derive mainly from the following reasons: first, it seems that they used some assumptions that were outdated and not reflective of current TAVI practice (relative survival reduction after TAVI vs. general population or short-term AEs rates). Second, it seems that they evaluated various relative treatment effects of TAVI (vs. SAVR) based on a combination of three RCTs: PARTNER 2, PARTNER 3, and Evolut LR. Treatment effects seem to be calculated from mixing studies indication (IR vs. LR), valve generation (SAPIEN XT vs. SAPIEN 3), interventional approach (TA vs. TF), and type of valve (BE vs. SE).

## 4. Discussion

The postgraduate school of health economics and management (ALTEMS) from Università Cattolica del Sacro Cuore has developed this analysis to assess the heterogeneity of the available HTA reports concerning TAVI on LR group over the last years. The authors of the manuscript tried also to analyze the existing gap to better understand the underlying reason of the disparity in the methodology assessments. Even though the evidence at the basis of the HTA evaluations included was quite homogeneous, the analysis highlighted some elements of heterogeneity. However, regarding the conclusions and recommendations contained in the reports, all HTA reports included in this analysis presented positive recommendations on TAVI in LR patients, considering it an effective and safe therapeutic alternative compared with SAVR. The main differences found concerned the type of evidence considered within the different studies included in the HTA reports, the methodology for assessing the quality of this evidence, and the fact that the data related to different generations of TAVI were not consistently differentiated. These limitations can partly delay a more widespread application of TAVI in clinical practice in those patients who can benefit from this technology. Moreover, some of the reports included information on low-risk patients and across all the surgical risk groups. This also posed limitations in the interpretability of the results on the safety and efficacy profile in low-risk patients. This distinction between surgical risks should be overcome since the latest ESC guidelines set an age over 75 years and the presence of transfemoral access as an indication for the use of TAVI. Regarding the heterogeneity of the economic impact, the models developed in the HTA reports define TAVI as either having a sustainable cost-effectiveness profile or a dominance profile. The results are also robust given the deterministic and probabilistic sensitivity analyses included in the economic evaluations analyzed. There is, however, a heterogeneity in the assumptions developed in economic evaluations. Considering that the evidence base appears to be very similar for the evaluations included despite the elements of heterogeneity described by our study, the question arises as to the role that HTA should play in evaluating the same health technology multiple times. The question arises for the following reasons. First, the recommendations of all HTAs are quite positive and agree on the need for long-term data to best characterize the final decision inherent in the uptake of TAVI for the low-risk population. Second, the drafting of a full HTA evaluation report requires a significant absorption both in terms of human and economic resources. Trying to avoid redundancies in the production of the HTA report could save resources that can be used to better characterize other unmet clinical need in the context in which the evaluation is performed. Furthermore, one of the aspects that is too often overlooked in HTA methodology is the process of updating the report once more evidence becomes available instead of developing another full evaluation. This is especially critical for medical devices such as TAVI that are often subject to incremental innovation over time. Thus, the timing of the re-assessment avoiding redundancies appears to be crucial. In this regard, to ensure equity of access to eligible patients while maintaining high standards of efficacy and safety, reference should be made to the lifecycle approach when evaluating technologies such as TAVI. This strategy involves engaging multiple stakeholders in early dialogs to reach agreements on outcomes in all HTA domains and incorporating pragmatic HTA methodologies that integrate RCTs, real-world evidence, and other robust clinical evidence, as well as innovative study designs. The primary objective was to define the lifecycle in a manner that optimally generates timely and relevant clinical evidence, as it tracks various products and product versions through development, the marketplace, and discontinuation. Post-market evidence generation can take diverse forms, but registries or other prospective observational studies are commonly used. Several factors unique to medical devices make registries an appropriate research methodology for post-marketing surveillance or informing payer decisions on pricing and reimbursement. These factors include uncertainty regarding long-term outcomes, as many medical devices are permanent implants; incremental design variations within product classes; the potential for significant heterogeneity in outcomes across populations due to patient, operator, and organizational factors; and extension of indications to other target populations. Typically, a registry accompanies the device to bridge evidence gaps across the total product lifecycle of the medical device (15). This approach should therefore simplify the decision-making processes related to the reimbursement of technology without neglecting a rigorous methodology to assess the efficacy and safety of medical devices. Also, within the lifecycle approach, it is important to define a clear indication on the timing of re-evaluation once new data on efficacy, safety, epidemiological burden, or economic evaluation become available in the literature through new registries, updates of clinical trials, clinical guidelines, etc. This would be relevant to standardize re-evaluation criteria, avoiding duplication in terms of HTA evaluations that lead to an inefficient allocation of available resources. Furthermore, to date, a solution to make more valuable the production of the assessments and have more time to focus on monitoring outcomes by HTA agencies can be represented by the recent EU regulation 2021/2282 on HTA (16) that introduced a joint clinical assessment that deals only with clinical domains. With the EU regulation, the aspects related to non-clinical impact (e.g., organizational or economic impact) areas and decisions on pricing and reimbursement remain at the national level. This could be helpful in order to have “centralized evidence” and full collaboration among the member states (MS) on the clinical domains. Furthermore, the EU regulation could be an opportunity to join the efforts of MS to develop shared registries to collect real-world data on the effectiveness of medical devices by facilitating access to innovation and equity of access for patients. In this way, regulatory agencies could avoid repeating full assessments by focusing on the domains of the HTA related to the economic, organizational, ethical, social, and legal impact (centralized clinical domains vs. country-specific domains). Nonetheless, in this particular setting, significant obstacles persist with regard to the role and acceptability of data from randomized trials and real-world sources, the balance between internal and external validity requirements, and the mandate for member states to utilize the clinical evaluation conducted by the EU HTA coordination group for local coverage and reimbursement decisions. Therefore, to address the limitations identified in our research, a comprehensive (lifecycle) approach that considers all the necessary evidence for a thorough evaluation of a given health issue should be recommended. Furthermore, in the field of medical devices, even subtle modifications to a device can have profound implications for clinical and safety outcomes. Thus, it is relevant to consider specific evidence on the type of technology that reflect current clinical practice in such a way as not to include evidence that may be outdated because it refers to previous generations of the technology. In conclusion, it would be useful to establish systematic methods for the dissemination of the HTA assessment and for the monitoring of their effective impact in orienting clinical practice identifying early warning systems to have enabling timely assessments useful to inform coverage and/or procurement both at national and local level. This can guarantee an agile communication of evaluation results to decision makers to speed up decision-making and avoid redundancies in the evaluations.

## 5. Conclusion

Several HTA evaluations have been developed to assess TAVI in patients at low surgical risk. At least five HTA reports on TAVI have been recently performed in the last years. Heterogeneity elements were found both in the quality of the evidence and the type of studies considered. However, HTA agencies reached the same recommendations on the use of TAVI. These redundancies in the information about TAVI have certainly placed limits on the interpretability of the results, delaying a more widespread use in the clinical practice. In our opinion, a centralized vision on the domains inherent to the efficacy and safety of medical devices, integrated by the individual members states with local studies and real-world data, and a local adaptation of the “soft domains” (economic, organizational, ethical, legal, and social), could have the potential to strengthen the role of HTA in Europe by ensuring faster decision-making and greater equity of access to health innovations. In addition to this, even the use of a lifecycle approach could simplify the decision-making processes related to the reimbursement of technology without neglecting a rigorous methodology to assess the comparative effectiveness and safety of medical devices. In this context, it is important also to underline the descriptive framework developed by Hutton et al. (17) which provides a comprehensive structure detailing reimbursement systems, with a distinction between policy implementation and technology decision levels. The policy implementation level encompasses the integration of the system within the broader political system, including establishment, objectives, implementation, and accountability. The technology decision level focuses on the process of individual reimbursement requests, involving assessment, decision-making, and implementation. Using this framework, information about reimbursement systems can be categorized into four research areas: constitution and governance, methods and processes, use of evidence, and accountability and transparency. Franken et al. (18), based on Hutton's framework, have introduced the concept of evaluation at the technology decision level, which quantifies the clinical, pharmacotherapeutic, and pharmacoeconomic value of a health technology, describing its quality and uncertainty of evidence. The appraisal of a health technology, on the other hand, aims to assess society's willingness to pay by considering assessment outcomes in relation to other societal criteria aligned with health system objectives. Thus, decision-making involves a value judgment from a broader societal perspective, taking into account health system objectives as well as non-healthcare-related goals. In order to enhance the legitimacy of societal decision-making processes, it is important for all systems to improve transparency, particularly in relation to the use and role of appraisal criteria in decision-making. Assessment and appraisal should be better distinguished from each other. It may be feasible to develop standardized European guidelines for evaluating clinical, and pharmacoeconomic evidence, especially since countries already consider evaluations from other countries that likely influence their own assessments, particularly in terms of clinical evidence. On the other hand, appraisal should remain specific to each country due to potential variations in social values. Reimbursement decisions for health technologies are inherently made in situations of uncertainty. While measures like risk-sharing schemes and temporary reimbursements have been introduced to mitigate the consequences of uncertainty and are gaining more attention, not all systems are currently equipped to systematically address uncertainty. Following reimbursement, the use of outcomes research and patient registries could improve the monitoring of real-world outcomes. Conducting comprehensive reviews of drug packages could enhance consistency in decision-making over time and improve overall value for money, thus ensuring sustainability. Moreover, countries could make better use of HTA to achieve value for money. HTA could play a greater role in systematically assessing and determining the level of added societal value and setting prices or reimbursement levels accordingly. Currently, countries only evaluate the performance of the system in terms of sustainability. It is recommended to develop tools that assess the impact of drug reimbursement on the other two objectives: quality of care and equity. Moreover, policymakers should reconsider the existing supply-driven system and explore the possibility of transitioning toward a more demand-oriented approach, in which they clearly state their willingness to pay for new health technologies that address unmet medical needs (18). Finally, this study contributes to the scientific debate in the light of the new EU HTA regulation. TAVI represents, in terms of HTA output, one of the most highly assessed medical devices among European and world agencies. The analysis comes at a crucial time since in the latest ESC guidelines the recommendations go beyond the surgical risk criteria, meaning that future evaluations will take all patients into account by considering other criteria such as age and feasibility of transfemoral access approach. In conclusion, evidence generation on TAVI should consider a lifecycle approach. This framework includes early multi-stakeholder dialogs to agree outcomes in all HTA domains and pragmatic HTA methodological approaches that integrate RCTs and real-world evidence in addition to other strong clinical evidence and innovative study designs. This approach seems to have the potential to reduce the heterogeneity in the assessment methods with the aim to ensure that all eligible patients who could benefit from TAVI treatment have adequate and timely access to innovation.
