# Supplementary material for: Analysis of heterogeneity of the different health technology assessment reports produced on the transcatheter aortic valve implantation in patients with severe aortic valve stenosis at low surgical risk
Source: Front Cardiovasc Med. 2023 Aug 10;10:1204520. doi: 10.3389/fcvm.2023.1204520 (PMC10450217; doi:10.3389/fcvm.2023.1204520)
Supplement: Supplementary file 1 [file Table1.docx]

**Appendix I: Main characteristics of the clinical trials used in the HTA report included**

| RCT | Current primary outcome Measures | Actual Enrollment | Detailed description |
| --- | --- | --- | --- |
| NOTION | Combined rate of death from any cause, myocardial infarction, and stroke [ Time Frame: 1 year]  Outcome measures will be defined as suggested by the Valvular Academic Research Consortium (VARC) | 280 | BACKGROUND: Transcatheter aortic valve implantation (TAVI) is a new and rapidly evolving treatment option for patients with severe degenerative aortic valve stenosis. Short- and mid-term results with transcatheter valve prostheses are promising in high-risk surgical patients, but long-term results are lacking. TAVI could potentially be an attractive minimally invasive treatment also for patients with moderate and low surgical risk, but no comparison has been made with the standard surgical treatment for aortic valve stenosis.  AIM: To compare TAVI and surgical aortic valve replacement (SAVR) in patients with severe aortic valve stenosis.  POPULATION: All patients with severe degenerative aortic valve stenosis referred for elective or subacute aortic valve intervention will be screened for study eligibility. To be included subjects must be 70 years or older, anatomical and technical eligible for both interventions, expected to survive more than 1 year after the intervention, and able to provide written informed consent. Study exclusion criteria include isolated aortic valve regurgitation or other significant valve disease, coronary artery disease requiring revascularisation at the time of referral, previous open heart surgery, a myocardial infarction or percutaneous coronary intervention within the last year, a cerebral infarction within the previous 30 days, severe renal -, pulmonary -, or infectious disease, and unstable preoperative condition.  DESIGN: The project is a national multicenter randomized clinical trial. Patients fulfilling all inclusion- and no exclusion criteria will be randomized to either TAVI or SAVR. Randomization will be 1:1 with 140 subjects in each group and stratified according to centre, age (70-74 years vs 75 and older), and coronary co-morbidity not requiring revascularisation (yes vs no). Primary outcome will be assessed by a blinded adjudication committee. Patients screened but not included in the study will be followed yearly. Screening and inclusion will commence in December 2009. Inclusion is expected to last 2 to 3 years, and subjects will be followed for 10 years.  INTERVENTIONS: Subjects randomized to TAVI will undergo percutaneous retrograde trans-femoral or trans-subclavian aortic valve implantation with the Medtronic CoreValve(TM) self-expandable bio-prosthesis. Before implantation a balloon dilatation of the aortic annulus will be performed. Subjects randomized to SAVR will undergo conventional surgical aortic valve replacement with a bio-prosthesis on cardiopulmonary bypass in normothermia with cold cardioplegia cardiac arrest. All interventions will be performed under general anaesthesia, and post-interventional medical and anticoagulation treatment will be uniform.  END POINTS: The primary end point is a combined outcome measure consisting of death from any cause, myocardial infarction, and stroke one year after the intervention. Secondary end points are death from any cause, cardiac death, cardiac -, cerebral -, pulmonary -, and renal complications, prosthesis re-intervention, procedure success and - time, admission lengths, functional class, quality of life, prosthesis and left ventricular structure and function. Follow-up visits will be performed after 30 days, 3, 6 and 12 months, and yearly thereafter for a minimum of 10 years. |
| STACCATO | The primary endpoint was the composite of all-cause mortality, cerebral stroke and/or renal failure requiring haemodialysis at 30 days. | NA | Aims: In a prospective randomised trial we aimed to compare transapical transcatheter aortic valve implantation (a-TAVI) with surgical aortic valve replacement (SAVR) in operable elderly patients.  Methods and results: The study was designed as a randomised controlled trial of a-TAVI (Edwards SAPIEN heart valve system; Edwards Lifesciences, Irvine, CA, USA) vs. SAVR. Operable patients with isolated aortic valve stenosis and an age ≥75 years were included. The primary endpoint was the composite of all-cause mortality, cerebral stroke and/or renal failure requiring haemodialysis at 30 days. After advice from the Data Safety Monitoring Board, the study was prematurely terminated after the inclusion of 70 patients because of an excess of events in the a-TAVI group. The primary endpoint was met in five a-TAVI patients (two deaths, two strokes, and one case of renal failure requiring dialysis) vs. one stroke in the SAVR group (p=0.07). In the a-TAVI group, one patient was converted to SAVR because of an abnormally positioned heart, and four patients were re-operated with open heart surgery because of annulus rupture (n=1), severe paravalvular leakage (n=2), and blockage of the left coronary artery (n=1). In the SAVR group, one patient was converted to TAVI because of a large intra-thoracic goitre.  Conclusions: Given the limitations of a small prematurely terminated study, our results suggest that a-TAVI in its present form may be associated with complications and device success rates in low-risk patients similar or even inferior to those found in high-risk patients with aortic valve stenosis. This will probably change in the near future with improved catheter based devices and better pre-procedural assessment. |
| PARTNER 3 | All-cause Mortality, All Stroke, and Rehospitalization (Valve-related or Procedure-related and Including Heart Failure) [ Time Frame: 1 year]  Number of patients that had any of these events | 1000 | Prospective, randomized, controlled, multi-center trial. Patients having an operative mortality < 4% (low operative risk) for surgical aortic valve replacement will be randomized 1:1 to receive either transcatheter heart valve replacement (TAVR) with the Edwards SAPIEN 3 or aortic valve replacement with a commercially available surgical bioprosthetic valve. Patients will be seen for follow-up visits at discharge, 30 days, 6 months, and annually through 10 years.  A subset of PARTNER 3 randomized patients will be enrolled in the Actigraphy/Quality of Life. Additional patients will be enrolled in either the Bicuspid Registry, Underrepresented Populations Registry (UPR) or the Alternative Access Registry. |
| EVOLUT LR | Safety: All Cause Mortality or Disabling Stroke Rate at 24 Months, Randomized Controlled Trial Safety: All Cause Mortality or All Stroke Rate at 12 Months, Continued Access Study [ Time Frame: Randomized Controlled Trial - 24 months Continued Access Study - 12 months]  Assessment of procedural safety by: All-cause mortality: all deaths from any cause after valve intervention. This includes all cardiovascular and non-cardiovascular deaths. Disabling stroke: a modified rankin score (mRS) of 2 or more at 90 days post-stroke and an increase of at least one mRS category from an individual's pre-stroke baseline. All stroke: any stroke after valve intervention (ischemic, hemorrhagic, or undetermined stroke). | 2223 | Multi-center, international, prospective, randomized, interventional, pre-market.  Subjects will be randomized on 1:1 basis to either TAVR with the Medtronic TAVR system or to SAVR. Patients will be seen at pre and post-procedure, discharge, 30 days, 6 months, 1 year, 18 months, and annually through 10 years.  The expanded use addendum is a multi-center, prospective, non-randomized continued access trial. All heart team approved subjects will be assigned to TAVR with the Medtronic TAVR system. Patients will be seen at pre- and post-procedure, discharge, 30 days, and annually through 10 years. Enrollment is expected not to exceed 3660 attempted implants in the United State |
