# Supplementary material for: Analysis of heterogeneity of the different health technology assessment reports produced on the transcatheter aortic valve implantation in patients with severe aortic valve stenosis at low surgical risk
Source: Front Cardiovasc Med. 2023 Aug 10;10:1204520. doi: 10.3389/fcvm.2023.1204520 (PMC10450217; doi:10.3389/fcvm.2023.1204520)
Supplement: Supplementary file 2 [file Table2.docx]

# Supplementary material

*Table S1 - Overview of the HTA reports included – Efficacy and Safety domains*

|  | CANADA | NORWAY | IRELAND | HAS (SAPIEN 3) | HAS (COREVALVE) |
| --- | --- | --- | --- | --- | --- |
| Inclusion criteria of the target population | Inclusion Criteria  Adults with severe aortic valve stenosis and low surgical risk  Surgical risk is defined by the study site’s multidisciplinary heart team informed by the Society of Thoracic Surgeons (STS) score and assessment of comorbidities. An STS score below 4% is generally considered low risk | Patients with severe aortic stenosis at high / intermediate / low surgical risk of death, as described by New York Heart Association Functional class (NYHA class), or by The Society of Thoracic Surgeons' risk model score (STS score), or European System for Cardiac Operative Risk Evaluation (EuroScore) or EuroSCORE II, with emphasis on studies reporting on low risk. | Patients with severe aortic stenosis (AS) at low or intermediate risk of death or complications associated with SAVR. | Patients with severe and symptomatic aortic stenosis older than 65 years, with a tricuspid orifice.  Patients with a life expectancy of less than 1 year due to extracardiac factors (co-morbidities) or with significant calcifications in the sub-aortic outlet chamber are not eligible for the technique (non-indication). | Patients aged at least 70 years at low risk (STS < 4%) with symptomatic severe native aortic stenosis (SVAoi < 0.5 cm²/m²) over the tricuspid orifice, without indication for mitral or coronary valve surgery (common trunk and/or SYNTAX > 32) and with anatomy favourable to the transfemoral route. The indication must be established during a multidisciplinary meeting, taking into account the risk scores and associated comorbidities. |
| Device | SAPIEN 3 (Partner3) CoreValve, Evolut R, and Evolut PRO (Evolut) | Devices with market approval | CoreValve*, Evolut R, Evolut PRO, SAPIEN 3 | EDWARDS SAPIEN 3 | COREVALVE |
| Study included | PARTNER 3 (mack 2019), The Evolut LRT (popma 2019) | PARTNER 3, The Evolut LRT, Staccato 2012 (IR and LR) and Notion 2015 (IR and LR) | Partner 2, Partner 2A, Surtavi, Staccato, Notion, Evolut LR 2019 and Partner 3 2019, GARY 2018, GARY 2019, Observant (registries) | Technology Assessment Report ONTARIO, PARTNER S3i, PARTNER 3 | Technology Assessment Report, HIQA, US COREVALVE, SOLVE-TAVI, FORWARD, NOTION, EVOLUT LOW RISK |
| Inclusion criteria of the evidence included | *Inclusion criteria:*  Randomized controlled trials and health technology assessments, systematic reviews, and meta-analyses of RCTs if they included the most recent RCTs in patients at low surgical risk identified both through the 2016 Health Quality Ontario HTA and their literature search. English language full-text publications *Exclusion criteria:*  Health technology assessments and systematic reviews if they did not include the most recent RCTs in patients at low surgical risk, non-randomized studies, noncomparative studies, editorials, commentaries, case reports, conference abstracts, and letters. Animal and in vitro studies | *Inclusion criteria:*  Inclusion criteria were adapted from the 2019 EUnetHTA report *Exclusion criteria:*  reviews published before April 2019 (before the publication of randomised trials on patients with low surgical risk) | *Inclusion Criteria:*  Randomised controlled trials. Real-world data derived from published studies from prospective national registries  *Exclusion criteria*:  Conference abstract, supplement or editorial, Emerging evidence or ongoing clinical trial, Posthoc subgroup analysis or Inappropriate study design, Inappropriate Intervention, Inappropriate population, Single arm TAVI registry, No TAVI device details, Inappropriate outcome, non-randomised international registry study, preliminary results. | *NA* | *NA* |
| STS Score | *Studies with an STS Score <4% have been included* | *Studies with an STS Score <4% have been included* | *Studies with an STS Score <4% have been included* | *Patients with an STS Score <4%* | *Patients with an STS Score <4%* |
| Safety & efficacy (conclusions) | TAVI was superior to SAVR on the basis of a composite end point of all-cause mortality, stroke, or rehospitalization at 1 year. The length of hospital stay was shorter for the TAVI than for the SAVR procedure. Patients in both the TAVI and SAVR groups experienced an improvement in symptoms and quality of life compared with before treatment. At 30 days, the degree of improvement for both outcomes were greater with TAVI than SAVR. The use of TAVI resulted in a lower risk of life-threatening or disabling bleeding, new-onset atrial fibrillation, and a slightly lower risk of acute kidney injury stages 2 or 3 compared with SAVR at 30 days. | (1) probably leads to little or no difference for the following short‐term outcomes: all‐cause mortality; stroke; myocardial infarction and cardiac death; (2) may reduce the risk of short‐term rehospitalisation, although the confidence interval also includes the possibility of no difference in risk between groups  (3) probably increases the risk of permanent pacemaker implantation. (4) reduces the risk of atrial fibrillation, acute kidney injury, and bleeding. (5) Uncertainty whether TAVI, compared with SAVR, affects the length of hospital stay in days, although it appears to be associated with shorter length of hospital stay. | TAVI was no less effective than SAVR in terms of all-cause and cardiac mortality based on follow-up data from 30 days to one year and was not associated with an increase in aortic valve reintervention. TAVI was found to be associated with reduced incidence of atrial fibrillation and life threatening or disabling bleeds. | Several randomized controlled trials specific to low-risk patients are available and demonstrate non-inferiority of transfemoral aortic valve bioprosthesis implantation over conventional surgery at 24 months on clinical endpoints minimizing all-cause mortality and stroke. These clinical outcomes are accompanied by a faster improvement in patients' quality of life and functional status while having a reduced length of stay and an almost systematic return home | Several randomized controlled trials specific to low-risk patients are available and demonstrate non-inferiority of transfemoral aortic valve bioprosthesis implantation over conventional surgery at 24 months on clinical endpoints minimizing all-cause mortality and stroke. These clinical outcomes are accompanied by a faster improvement in patients' quality of life and functional status while having a reduced length of stay and an almost systematic return home |
| Primary objective | The objective of this analysis was to explore the underlying values, needs, preferences and priorities of people who have lived experience with aortic valve stenosis and those having experience with transcatheter aortic valve implantation (TAVI) or surgical aortic valve replacement (SAVR). The treatment focus was TAVI versus SAVR. | Update and summarise current knowledge on effectiveness and safety with transcatheter aortic valve implantation/ replacement (TAVI) compared with surgical aortic valve replacement (SAVR) in the treatment of patients with severe aortic stenosis across surgical risk groups, including patients with severe aortic stenosis and high surgical risk. | The purpose of this health technology assessment (HTA) is to provide advice to the Minister for Health, the Department of Health and the Health Service Executive (HSE) on the implementation of a transcatheter aortic valve implantation (TAVI) pathway in the public health care system for patients with severe symptomatic aortic stenosis at low or intermediate risk of surgical complications. The Health Information and Quality Authority (HIQA) agreed to undertake the HTA following a formal request from the HSE. | Evaluation of the therapeutic effect/adverse effects, risks related to the use | Evaluation of the therapeutic effect/adverse effects, risks related to the use |
| Clinical guidelines included | AHA/ACC focused update of the 2014 AHA/ACC guideline for the management of patients with valvular heart disease: a report of the American College of Cardiology/American Heart Association task force on clinical practice guidelines. | AHA/ACC Focused Update of the 2014 AHA/ACC Guideline for the Management of Patients with Valvular Heart Disease: A Report of the American College of Cardiology/American Heart Association Task Force on Clinical Practice Guidelines.  Transcatheter or surgical aortic valve replacement for patients with severe, symptomatic, aortic stenosis at low to intermediate surgical risk: a clinical practice guideline. | AHA/ACC Focused Update of the 2014 AHA/ACC Guideline form the Management of Patients with Valvular Heart Disease. Journal of the American College of Cardiology.  2015 ESC Guidelines for the management of infective endocarditis: The Task Force for the Management of Infective Endocarditis of the European Society of Cardiology (ESC). Endorsed by: European Association for CardioThoracic Surgery (EACTS), the European Association of Nuclear Medicine (EANM).  Guidelines on the management of valvular heart disease (version 2012) The Joint Task Force on the Management of Valvular Heart Disease of the European Society of Cardiology (ESC) and the European Association for Cardio-Thoracic Surgery (EACTS). European  AHA/ACC Guideline form the Management of Patients with Valvular Heart Disease. Journal of the American College of Cardiology | None | None |
| Recommendations | Ontario Health, based on guidance from the Ontario Health Technology Advisory Committee, recommends publicly funding transcatheter aortic valve implantation in adults with severe aortic valve stenosis who are at low surgical risk | Further to the NIPH report, the published decision from the Decision Forum of RHAs (Regional Health Authorities) concluded that across all risk groups, catheter-based implantation of aortic valves can be used in the treatment of patients with severe aortic stenosis in hospitals already performing cardiac surgery | The extension of the TAVI care pathway to include patients with severe symptomatic aortic stenosis at low and intermediate surgical risk should be considered in the Irish public healthcare system. Despite the available evidence are characterized by a low and moderate certainty of evidence, they showed no difference in efficacy outcomes between TAVI and SAVR. | The HAS recommended positively the reimbursement of SAPIEN for all patient groups. | The HAS recommended positively the reimbursement of SAPIEN for all patient groups |

*Table S2 - Overview of the HTA reports included – Economic domain*

|  | CANADA | NORWAY | IRELAND | FRANCE (SAPIEN 3) | FRANCE (COREVALVE) |
| --- | --- | --- | --- | --- | --- |
| Type of model | Markov model  Budget impact | Markov model  Budget impact | Probabilistic Markov model, except Kaier 2019 using Secondary data analysis  Budget impact | Markov model  Budget impact | Markov model  Budget impact |
| Study design | CUA (literature review), BIM | CUA, BIM | CUA, BIM | Cost-utility analyses and Cost-effectiveness analysis | Cost-utility analyses and Cost-effectiveness analysis |
| Perspective | Health System perspective | Health System perspective | Health System perspective | Health System perspective | Health System perspective |
| Time horizon | Life time horizon (15 years)  BIM (5 years) | 15 years (lifetime) time horizon  BIM (5 years) | Baron 2019, Tam 2018a, Tam 2018b, Zhou 2019 life time horizon, Goodall 15 years horizon Kodera 10 years horizon  BIM (5 years) | 15 years (lifetime) time horizon  BIM (5 years) | 15 years (lifetime) time horizon  BIM (5 years) |
| WTP threshold | Two WTP threshold has been taken into account: 50,000$ / QALY 100,000$ / QALY | Officially defined willingness to pay (WTP) threshold | Kodera WTP (43,500$), Tam WTP 50.000 $ Goodall (France) no WTP stated, Zhou WTP 50,000 $, Baron WTP 50,000 $ | 50.000€/QALY | 80.000€/QALY |
| Device type | Balloon-expandable and self-expandable TAVI | SAPIEN 3 (Partner 3 trial) | SAPIEN 3 (Partner 3 trial) | SAPIEN 3 | CoreValce Evolut R/ Evolut PRO |
| Discount rate | 1,50% | 4% | Baron 3%, Goodall 4%, Kodera 2%, Tam 1,5%, Zhou 5% |  |  |
| Main assumptions | The average age of the cohort in the cost-utility analysis was 73,3. The model considered the following health states: Alive/well, disabling strokes, >moderate PVL and death.  Complication rates occurring 1 year or longer after the procedure were the same for TAVI and SAVR Mortality rates were equivalent to age- and gender-specific Canadian life tables. No formal public funding for TAVI in patients at low surgical risk | Patients are 73 years of age when entering the model. The Markov model considered 3 health states: alive and well, post major complications and death. The model is based on data at only one-year follow up and long-term studies on survival, procedure-related complications, prostheses’ longevity (used both in TAVI and SAVR). All complications are treated independently. Budgetary consequences of extending TAVI as a routine treatment onto patients with severe aortic stenosis and lower risk groups. The uptake of TAVI will continue to rise at the rate of between 10 and 20% annually. | All patients aged 70 years or older requiring isolated aortic valve replacement would be treated with TAVI.  The Markov model considered the following health states: Alive/well, major complications (acute kidney injury, disabling stroke, myocardial infarction), post major complications, hospitalizations and death. No capacity constraints for cath lab and additional procedures. Postoperative complications were captured in the trial data  Mortality has been compared to the general population | The model cohort age was 73.  - the modelling is based on health states defined as “Alive and well”, “Disabling strokes”, “Treated Atrial fibrillation” and “Death”  - the use of relative mortality risks from non-SAPIEN 3 specific studies and extrapolation assumptions based exclusively on literature validated by the Scientific Committee established for this economic analysis. | The model cohort age was 65+. The modelling is based on health states defined as “no stroke”, “stroke”, “post stroke” and “death”. It was assumed that only living patients without a stroke can have a stroke: patients who have already had a stroke cannot have a new stroke.  In the baseline analysis, for year 1 (2021), based on market knowledge, it was assumed that patients (a % of the target population) could benefit from TAVI, cause the reimbursement for TAVI in this indication is unlikely to occur in a full year. |
| Results (ICER and BI) | ICER (BE TAVI vs. SAVR): $27,196/QALY (dominant per 50,000$ e 100,000$) ICER (SE TAVI vs. SAVR): $59,641/QALY (dominant per 100,000$)  We estimate that the additional cost to provide public funding for TAVI in people with severe aortic valve stenosis at low surgical risk would range from about $5 to $8 million over the next 5 years. The budget impact publicly funding TAVI in Ontario is estimated to be an additional $5 to $8 million each year for the next 5 years. The budget impact could be significantly reduced with reductions in the device price. | Tavi has been shown be dominant with an incremental cost of -35,283 (NOK) and an incremental effect (QALY) of 0.054.  The budget impact analysis indicates that the introduction of TAVI for low-risk patients is likely to be cost-neutral in the short run. We have not accounted for the costs of the capacity expanding. | Over a five-year period, TAVI is estimated to save €0.1 million (95% CI: €-3.1 to  €2.9 million) compared with SAVR, which therefore may be considered budget  neutral. The estimated budget impact is based on treating 100 patients each  year, comprising 67 low and 33 intermediate surgical risk patients. | It results in the dominance of valve replacement surgery by SAPIEN 3. According to the proposed modelling, this strategy would save four months of life and induce a cost reduction of €7,737 over a 15-year time horizon. Regarding the budget impact, at the claimed price of €17,175.40, the introduction of SAPIEN 3 results in a reduction in health insurance expenditure of around €67M over 5 years. | At the manufacturer's asking price of €15,419.21 resulted in:  - an ICER of €7,571.39/ALY and €5,893.01/QALY compared with aortic valve replacement surgery.  When the stated price of CoreValve Evolut R/PRO falls by 5% or 10%, surgery is dominated by CoreValve Evolut R/PRO.  At an asking price of €15,419.21, the impact on the budget is €63,679,321 over 5 years. |
| Sensitivity analyses | In probabilistic sensitivity analysis, balloon-expandable TAVI had the highest probability of being cost-effective; it was the preferred option in 53% and 58% of model iterations, at willingness-to-pay values of $50,000 and $100,000 per QALY, respectively. Self-expanding TAVI was preferred in less than 10% of iterations.  The greatest increase in budget impact is when there is the introduction of TAVI would lead to an expansion of the target population and the scenario where SAVR procedure costs were reduced by 10%. The greatest decrease in budget impact is in the scenario with a gradual uptake rate from 20% to 50% over 5 years. | The results of sensitivity analysis of our model analysis showed that cost parameters related to the TAVI procedure had the greatest impact on the results.  The budget impact analysis based on the results of the cost-effectiveness analysis, and some conservative assumptions about expansion in the use of TAVI indicates that the incremental annual total cost of this expansion will reach 32.5 million Norwegian kroner in the course of five years.” Only two of these parameters may influence cost-effectiveness to the degree that willingness to pay for health potentially could matter for the decision, namely if TAVI is 30% more costly than assumed, and if SAVR is 30% less costly than assumed. | WTP threshold has been assumed to 20.000 $. The parameters creating the most sensitivity is the cost of SAVR procedure [€24,120 to €35,696] and Cost of TAVI procedure [€22,833 to €33,792] in low-risk patient. The cost-effectiveness of TAVI was mainly affected by the cost of the TAVI and SAVR procedures. At the lower and higher cost estimate for the SAVR (€24,120) and TAVI (€33,792) procedure, respectively, TAVI was no longer cost-effective in intermediate and low risk patients, assuming a WTP threshold of €20,000 per QALY gained. | None of the parameters tested in the deterministic (univariate) sensitivity analysis changed the conclusions of the baseline analysis, with the exception of the use of the upper bound on the probability of transition in the SAPIEN 3 arm from "alive and well" to "disabling stroke" beyond the 2-year period (RDCR = €2,214/QALY). Apart from this extreme case, the results are robust and the RDCRs are all negative (dominance situation).  The probabilistic analysis shows that SAPIEN 3 is the dominant strategy in 70% of the cases for a willingness to pay of 0 €/QALY. This probability approaches 100% as soon as the willingness to pay reaches €40,000/QALY.  The price below which SAPIEN 3 is cost-effective at the threshold of €50,000 per QALY (all else being equal) is €54,900 and the equilibrium price below which SAPIEN 3 is dominant, regardless of the threshold, is set at €24,500. | The main factor inducing a very high variability of the ICER is the stroke rate observed in the clinical trial for the surgical strategy: the variation of this parameter makes the RDCR vary from 42 678 €/QALY to -31 037 €/QALY (the surgical strategy is dominated). No scenario sensitivity analysis shows that shows that CoreValve Evolut R/PRO is dominated by surgery.  The main scenario sensitivity analyses that lead to high variability in ICER are those on variation in time horizon and method of assessing hospitalization costs.  For probabilistic sensitivity analyses, the willingness-to-pay (WTP) for which CoreValve Evolut R/PRO has an 80% probability of being cost-effective compared with surgery is 80,000/QALY. |
| Data used | Literature review  Tam DY, Azizi PM, Fremes SE, Chikwe J, Gaudino M, Wijeysundera HC. The cost-effectiveness of transcatheter aortic valve replacement in low surgical risk patients with severe aortic stenosis. Eur Heart J Qual Care Clin Outcomes [Internet]. 2020 Jul 9 [cited 2020 Aug 20]; doi: 10.1093/ehjqcco/qcaa058. Available from: https://academic.oup.com/ehjqcco/advance-article-abstract/doi/10.1093/ehjqcco/qcaa058/5869438?redirectedFrom=fulltext | All transition probabilities that inform the model were derived from data for clinical  outcomes at 30-days and 1-year from the randomised controlled multicentre trial  PARTNER 3 (Placement of Aortic Transcatheter Valves 3) | The available evidence was often based on first generation devices that no longer commercially available, such as CoreValve™ and SAPIEN in intermediate risk patients and Evolut™ R in low-risk patients. These have largely been replaced in clinical practice by newer devices, such as SAPIEN XT, SAPIEN 3, and Evolut R. For the purposes of the economic model, only evidence on devices currently used in clinical practice in Ireland was used, as detailed below for each patient group.  Low risk  Two clinical trials, to date, have evaluated the clinical effectiveness and safety of TAVI compared with SAVR in patients at low surgical risk: EVOLUT Low risk and PARTNER 3 | PARTNER 3 clinical trial | The pivotal Evolut Low risk clinical trial was considered. |
| Conclusions | The TAVI procedure might be cost-effective for patients at low surgical risk (more QALY and more expensive); however, there is some uncertainty in this result. | TAVI for patients at low surgical risk is slightly more effective (0.05 QALYs gained) and less costly (saving of NOK 35 000) than SAVR | Compared with SAVR, TAVI is considered a highly cost-effective treatment option for patients aged 70 years and over at low or intermediate surgical risk | The analysis of the efficiency of the SAPIEN 3 aortic valve for the management of patients with severe aortic stenosis at low surgical risk in France shows a dominant RDCR in the baseline analysis. Over a time horizon of 15 years and considering updated results:  - the number of QALYs gained with SAPIEN 3 compared to surgery is 0.64 (7.49 QALYs versus 6.85) and the number of life years gained with SAPIEN 3 is 0.33 (10.17 years versus 9.84).  - The incremental cost saving with the use of SAPIEN 3 compared to surgery is estimated at €7,737 per patient (€40,855 versus €48,592). | At the manufacturer's asking price of €15,419.21 resulted in:  - an ICER of €7,571.39/ALY and €5,893.01/QALY  The market introduction of TAVI in patients at low risk for aortic valve replacement (STS score <4%) results in an increase of 64 million over 5 years.  This budget impact estimate is also characterised by a high degree of uncertainty related to market share estimation due to the period in which the data were collected, the patients covered by TAVI in this period and the absence of information on the market share distribution among TAVI from 2023 onwards. |
